# Moving Forward in Space and Time: How Strong is the Conceptual Link between Spatial and Temporal Frames of Reference?

**DOI:** 10.3389/fpsyg.2012.00486

**Published:** 2012-11-15

**Authors:** Andrea Bender, Annelie Rothe-Wulf, Lisa Hüther, Sieghard Beller

**Affiliations:** ^1^Department of Psychology, University of FreiburgFreiburg, Germany; ^2^Center for Interdisciplinary Research, Bielefeld UniversityBielefeld, Germany; ^3^Department of Human Sciences, University of PaderbornPaderborn, Germany

**Keywords:** frames of reference, space, time, cross-linguistic comparison (German, English, Chinese, Tongan), dynamic settings

## Abstract

People often use spatial vocabulary to describe temporal relations, and this increasingly has motivated attempts to map spatial frames of reference (FoRs) onto time. Recent research suggested that speech communities, which differ in how they conceptualize space, may also differ in how they conceptualize time and, more specifically, that the preferences for spatial FoRs should carry over to the domain of time. Here, we scrutinize this assumption (a) by reviewing data from recent studies on temporal references, (b) by comparing data we had collected in previous studies on preferences for spatial and temporal FoRs in four languages, (c) by analyzing new data from dynamic spatial tasks that resemble the temporal tasks more closely, and (d) by assessing the co-variation of individual preferences of English speakers across space and time. While the first set of data paints a mixed picture, the latter three do not support the assumption of a close link between referencing preferences across domains. We explore possible reasons for this lack of consistency and discuss implications for research on temporal references.

## Introduction

Space and time are closely linked – not only in physics, but also in lay people’s descriptions and conceptualizations, and maybe even in the computational mechanisms of the brain. For instance, when we talk about time, we tend to use spatial vocabulary (e.g., Clark, 1973; Bennett, 1975; Traugott, 1975, 1978; Miller and Johnson-Laird, 1976). When we reason about time, temporal representations may be affected by spatial primes (Boroditsky, 2000, 2001; Gentner et al., 2002), by spatially defined response modes (Torralbo et al., 2006; Weger and Pratt, 2008), or by primes based on imagined or fictive motion (Boroditsky and Ramscar, 2002; Matlock et al., 2005). Moreover, time, space, and quantity appear to be part of a generalized magnitude system (Walsh, 2003), and temporal relations tend to be mapped onto and to be computed in terms of spatial representations (Casasanto and Boroditsky, 2008; Casasanto et al., 2010).

Consequently, speech communities that differ with regard to how they conceptualize space should also differ in their conceptualization of time. A promising way of assessing differences in spatial conceptualization is by assessing preferences in frames of reference. A frame of reference (FoR) is a coordinate system required to describe the relation between objects from a given perspective. The taxonomy proposed by Levinson (2003) distinguishes three main types – absolute, intrinsic, and relative – and speakers of different languages have been shown to differ with regard to which FoRs they habitually and/or preferentially use (Senft, 1997; Bennardo, 2002; Levinson, 2003; Majid et al., 2004; Haun et al., 2006, 2011; Dasen and Mishra, 2010). Whether these distinct preferences also entail cognitive implications is a matter of on-going dispute (Levinson et al., 2002; vs. Li and Gleitman, 2002; and see Haun et al., 2011; Li et al., 2011). The question we are interested in is whether these preferences for a specific FoR in the spatial domain carry over to the temporal domain and, if so, how strong this conceptual link is.

## Cultural Variability in Space-Time Mapping

Recent attempts to systematically map taxonomies of spatial FoRs onto the temporal domain yielded a variety of accounts (e.g., Bender et al., 2005, 2010; Kranjec, 2006; Moore, 2006, 2011; Núñez et al., 2006; Zinken, 2010; Tenbrink, 2011; Yu, 2012), but are far from converging. In line with these theoretical disputes, empirical studies also paint a mixed picture.

Usage of an absolute FoR in time (with past in the East and future in the West), for instance, has been observed in card arrangement tasks by members of a Pormpuraaw Aboriginal speech community speaking Kuuk Thaayorre, who also prefer the absolute FoR to organize spatial representations (Boroditsky and Gaby, 2010). Likewise, the Yupno in Papua New Guinea prefer an absolute FoR in both spatial and temporal descriptions, indicating past events by downhill gestures, and future events by uphill gestures (Núñez et al., 2012). Matters are more complicated for Tzeltal Maya speech communities, which prefer an absolute FoR (along the downhill/uphill axis) for spatial descriptions. Occasionally, they also equate uphill with the future, however less consistently so (Brown, 2012).

The concern that spatial FoRs *per se* may not be the only relevant factor for temporal references is also indicated by findings that establish strong correlations between the prevailing writing direction^1^, and a temporal representation in form of a mental time line: left to right in English speakers, right to left in Hebrew and Arabic speakers (Tversky et al., 1991; Fuhrman and Boroditsky, 2010), and top-down in Chinese speakers (Boroditsky et al., 2011; Bergen and Chan Lau, 2012).

The primacy of space as the source domain for conceptualizing time has been disputed more generally on other grounds as well. The claim, for instance, that speakers of Mandarin Chinese make more frequent use of vertical spatial *metaphors* for time than English speakers and are therefore more likely to also think about time in a vertical manner (Boroditsky, 2001), gave rise to an on-going debate (for disconfirmation, see Chen, 2007; January and Kako, 2007; Tse and Altarriba, 2008; for confirmative evidence, see Boroditsky et al., 2011; Fuhrman et al., 2011; Miles et al., 2011), which has not been settled yet (see the review by Chen and O’Seaghdha, in press). Speakers of Yucatec Maya, who are habitual users of an absolute FoR in space and who refer to locations and directions by precise (horizontal) gestures (Le Guen, 2011), *avoid* mappings of temporal entities onto any of these horizontal locations and directions; instead they tend to point toward the ground for the here and now and toward the sky for distant past or future events (Le Guen and Pool Balam, 2012). In the case of Aymara, the question of what one can know (due to personal experience) seems to provide the basic motivation of a front-to-past mapping (Núñez and Sweetser, 2006). And the Amazonian Amondawa are reported to completely lack space-time mappings even at the constructional linguistic level (Sinha et al., 2011). These studies lend support to theoretical claims (e.g., by Galton, 2011) that not all attributes of time can be mapped onto space, and that some speech communities may entirely refrain from relating their temporal conceptions to spatial ones.

Even in cases, where space-time mappings were observed, they need not be mediated by a straightforward linguistic mapping. Thaayorre, for instance, do not *speak* of the future as “westwards” (Gaby, 2012). Yupno has isolated expressions with overlapping spatial and temporal meanings, but not in a systematic manner (Núñez et al., 2012). And Tzeltal provides a wide range of spatial expressions that can be mapped onto time, thus giving rise to a wide range of temporal representations, as reflected in responses to the card arrangement task mentioned above (Brown, 2012). The cases of Kuuk Thaayorre and Yupno therefore provide support for the assumption that a specific FoR (here: the absolute FoR) may be transferred from space to time – solely or primarily on the basis of the underlying *principle* (here: by deriving orientation from the superordinate field).

Tzeltal and Amondawa, on the other hand, indicate that such a transfer of principles need not be the case. Given the incomplete linguistic correspondence across domains, however, these languages cannot be taken as evidence against a stringent mapping of spatial FoRs onto temporal contexts. A stronger case for investigating the transfer of FoR preferences across domains would be provided by languages that do contain similar expressions for spatial and temporal sequencing. In other words, if front for these expressions were assigned in time according to the same principle as it is assigned in space (i.e., with the same FoR), then one could safely assume a strong conceptual link between spatial and temporal representations. A paradigmatic task that has been used to scrutinize this link is the Wednesday’s meeting task, as will be explained in the next section.

## Moving Forward: Temporal References in Dynamic Settings

When confronted with the question “Next Wednesday’s meeting has been moved forward 2 days. What day is the meeting now?” roughly half of USA-American participants respond with Friday, the other half with Monday (e.g., McGlone and Harding, 1998).

### Accounts of the ambiguity in “moving forward”

The ambiguity inherent in the “moving forward” expression has been attributed to the fact that time can be conceptualized by adopting one of two perspectives (Clark, 1973; McGlone and Harding, 1998; Evans, 2003): the *Moving Ego* (*ME*) *perspective* takes Ego as approaching future events and leaving them behind; the forward-movement would thus be interpreted as futurewards (i.e., to Friday). The complementary *Moving Time* (*MT*) *perspective* takes future events as approaching Ego and passing by; the forward-movement would thus be interpreted as pastwards (to Monday). These perspectives can be primed not only by temporal, but also by spatial stimuli (McGlone and Harding, 1998; Boroditsky, 2000; Boroditsky and Ramscar, 2002; Gentner et al., 2002), indicating a conceptual link between spatial and temporal representations.

Alternatively, people’s readings of the “moving forward” expression can also be explained from a theoretical perspective that focuses on *temporal FoRs* analogous to the FoRs used for space (cf. Bender et al., 2010; Rothe-Wulf et al., under review). From this perspective, the ambiguity of “moving X forward” arises from the fact that this expression is inherently underspecified: in order to determine the direction of the forward-movement, one has to assign a front to the constellation – both in space and time – but the section, to which front is assigned, depends on the adopted FoR, again both in space and time (see also Moore, 2011).

**Figure 1:**
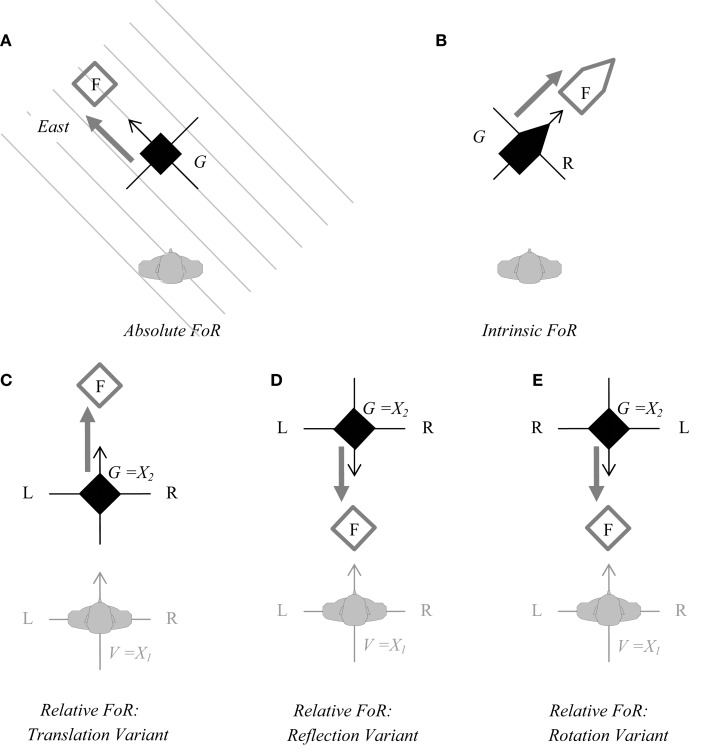
**Moving the object “forward” from its original position (G) toward the new position (F) according to different frames of reference (FoRs): the absolute FoR (A), the intrinsic FoR (B), and the three variants of the relative FoR (C), (D), and (E)**. *Note:* The array is depicted from above. G is colored black, F white, and the observer gray (gaze direction is indicated by the tip of the nose). The thick gray arrow indicates the movement of F from is original position G to the new position. Left is indicated by L, right by R, the *origo* of the coordinate systems by *X*, and their (acquired) FRONT by the tip of the thin arrow. In the relative FoRs, the primary coordinate system (*X*_1_) originates in Ego, the secondary coordinate system (origo *X*_2_ = G) is obtained **(C)** by translation into G, **(D)** by reflection in G, or **(E)** by rotation in G.

Typically, spatial FoRs have been described for static settings (e.g., Levinson, 2003). However, they can easily be transferred to dynamic descriptions while largely retaining their structure. As in static settings, the main relation to be established in dynamic settings is that between a figure F and a ground G (in reference to which F is located). The only difference is that, whereas in static settings F and G are two distinct entities, in dynamic settings G is the original position of the entity, and F is the position to which this entity is moved (cf. Figure 1).

The absolute FoR (Figure 1A) may be the least likely to be associated with expressions of “moving forward,” as it typically involves bearings that are linked to geographical landmarks like cardinal directions or the uphill/downhill gradient. In some cases, however, one of these geographical bearings is privileged and may thus become the front of the superordinate field. In some cultural contexts, for instance, this is East (as the very term “orientation” indicates), in others it is the direction in which Mecca is located, and for the Aymara it is where the sun rises (Núñez and Cornejo, 2012). Another option for assigning front in an absolute FoR is described by Talmy (2000): when entities are part of a sequence, like people waiting in a queue, the whole sequence can be seen as an “encompassive secondary reference object” (in contrast to the single entities which are conceptualized as the “primary reference object”) and are treated, in some accounts, as the field for an absolute FoR. In this case, front is derived from alignment in the sequence and/or moving direction, which overrides the (possible) orientation of the single entities (Talmy, 2000).

The two basic FoRs that are more typically invoked by “forward”-expressions are the intrinsic FoR and the relative FoR, and they are distinguished by whether or not the viewpoint of an observer (V) is also considered. For the *intrinsic FoR* (Figure 1B), this viewpoint is irrelevant; however, the FoR can only be adopted if the object to be moved has an intrinsic front already assigned to one side (e.g., the front of a car). front and forward motion are then projected onto the section of space pertinent to this side (i.e., a car’s canonical driving direction).

Under a *relative FoR*, assignment of front is derived from V (i.e., the observer’s face). How this front is then projected onto the object to determine the direction of its forward motion depends on which variant of the relative FoR the speaker chooses: translation, reflection, or rotation. In the case of *translation*, front and forward motion are projected in gaze direction of V onto the space beyond G (Figure 1C), in the case of *reflection* and *rotation*, they are projected onto the space between V and G (Figures 1D,E). The distinction of reflection and rotation requires the left-right axis, which has no temporal counterpart; for this reason, the reflection and rotation variant will be collapsed in the following. For more detailed descriptions, see also Beller et al. (under review) and Levinson (2003).

Crucially, this taxonomy of FoRs holds regardless of whether the constellation to be described is a *spatial* array of objects (Levinson, 2003; Beller et al., under review) or a *temporal* array of events (cf. Bender et al., 2010; Rothe-Wulf et al., under review), allowing for the analysis of whether the preferred temporal reading of “moving forward” reflects the preferred spatial reading within a speech community (cf. Table 1).

**Table 1:** **Direction of “forward” in dynamic settings depending on the FoRs in space and time (with G referring to the ground object)**.

FoR	Abstract principle	In space	In time	
			Past events	Future events
Absolute	Into the direction of the superordinate field	FRONT of the (spatial) field (e.g., east/eastwards)	FRONT of the (temporal) field: the arrow of time = futurewards	
Intrinsic	Into the direction of G’s FRONT	G’s (spatial) FRONT	G’s (temporal) FRONT: *before* its beginning = pastwards	
Relative: translation	Away from the deictic center (=further)	Away from observer V (=further)	Away from now (=further) =pastwards	Away from now (=further) =futurewards
Relative: reflection (rotation)	Toward the deictic center (=nearer)	Toward observer V (=nearer)	Toward now (=nearer) =futurewards	Toward now (=nearer) =pastwards

The characterization of the *absolute* FoR as depicted here depends on whether “front” and “forward” can be defined for the superordinate field (outside figure, ground, and observer). In the *spatial* domain, this is most often not the case (as in English, where cardinal directions are used instead). For the Aymara, however, Eastwards is the privileged orientation of the spatial field (Núñez and Cornejo, 2012), and may thus afford a “forward” direction. In contrast, matters are less complicated for the *temporal* domain, as the directionality of time itself provides this orientation. Most languages under scrutiny here take the arrow of time as pointing toward the future, and this is where front is assigned to. Events “in front of” other events or “moved forward” from their previous position would thus be further in the future under an absolute temporal reading (for the reversed conception of time in Malagasy, Toba, and Aymara, in which front is assigned to the past, see Klein, 1987; Dahl, 1995; Núñez and Sweetser, 2006, respectively).

An *intrinsic* FoR, in contrast, derives its orientation from the ground entity G (events in the temporal domain), whose intrinsic front is their beginning: front is thus assigned to the time before the beginning of event G. Accordingly, events “in front of” other events or “moved forward” from their previous position would be in the past of the original date.

A *relative* FoR, finally, requires a ternary relation between figure F, ground G, and observer V. Crucially, it emerges as either one of two different (and in fact opposed) variants: in the *reflection* variant, front is assigned to the time between G and V (i.e., nearer to V), whereas in the *translation* variant, front is assigned to the time beyond G (i.e., further away from V). In either case, events are localized symmetrically in one’s past and future, and thus with diverging fronts and backs.

### Investigation of FoRs across domains: A re-analysis of previous findings

In two previous studies we had assessed which spatial FoRs (s-FoRs) speakers of German, USA-English, Mandarin Chinese, and Tongan use for the description of relationships between objects (Beller et al., under review), and which temporal FoRs (t-FoRs) speakers of these languages use for moving an event (Bender et al., 2010). In the spatial tasks, participants were presented with 12 depictions of spatial layouts, and were asked to identify the position of F in reference to G. In the temporal tasks, four events were described that had been moved forward, either in the past or in the future. They were then asked to specify the date or time, to which the event had been moved. Both for the spatial and the temporal tasks, responses were categorized in terms of FoRs according to the above described principles. In almost all cases, different FoRs are preferred for spatial than for temporal descriptions (see Table 2).

**Table 2:** **Most frequently adopted FoRs in the four investigated countries for space (Beller et al., under review) and time (Bender et al., 2010)**.

Domain	Country			
	Germany	USA	China	Tonga
Space	Reflection	Reflection	Translation	Translation
Time	Intrinsic	Absolute/intrinsic	Intrinsic	*No clear* *preference*

May this incongruence be taken as strong evidence against a (close) link between spatial and temporal references, and thus indicate incongruence across domains, or could it otherwise be accounted for?

The principle according to which we classified the response patterns in the temporal tasks as temporal FoRs were derived from a thorough conceptual analysis for *future* events (or, more precisely, for events regarded as *in front of* speakers). For past events, however, the classification rests on the assumption that people do re-orient to events in their back by way of rotation^2^ (Bender et al., 2010). This assumption authorizes the point-symmetric pattern for future and past responses proposed here (e.g., the diagnosis of a reflection variant of the relative FoR if events both in the past and the future are “moved forward” toward the present; cf. Table 1). Whether this rotation assumption really holds in the temporal domain needs to be scrutinized more thoroughly in light of new findings on dorsal references (i.e., for spatial arrays in one’s back), for which rotation was not observed (at least not in the settings examined in Beller et al., under review).

Bearing these uncertainties in mind, we re-classified the responses people gave in our previous experiments according to whether the entities were moved *away from* or *toward* the observer. As we wanted the spatial and the temporal tasks to be as similar as possible (i.e., with the relevant entities all arranged along one dimension) and to be independent of the rotation assumption, we considered for re-analysis only those two spatial layouts from the data reported in Beller et al. (under review), in which figure F and ground object G were arranged in one line with the observer and in which the objects were in the observer’s visual field. We then computed the mean frequency of assigning front to the side of G that was oriented either away from (further) or toward the observer (nearer). From the temporal data reported in Bender et al. (2010), we considered only those tasks in which the movement took place in the observer’s subjective future (so as to avoid the question of observer rotation), and we classified this movement as either futurewards (further away from the present) or pastwards (nearer to the present).

**Figure 2:**
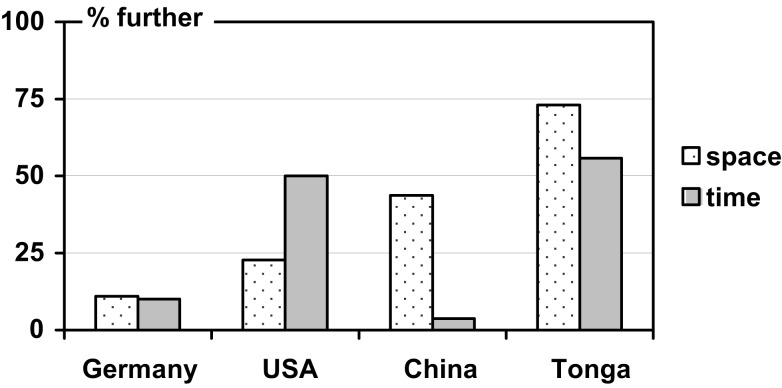
**Percentage of individuals assigning FRONT *further away* from the observer in space and time (cf. Table 3)**.

While these two readings (further/nearer) can be directly generated from the FoRs in Table 2, they are less discriminative than the FoRs. Interestingly, though, consistency across domains increased only slightly by this recoding (see Table 3 and Figure 2). A consistent pattern with a strong preference for assigning front in the same direction across both the spatial and temporal tasks was detected only in one of the four languages (German), while in the other three languages a predominance of one FoR either in the spatial or temporal tasks was paired with a mixed assignment of front (around 50%) in the other task, respectively^3^.

**Table 3:** **Percentage of individuals assigning FRONT either *further away from* or *nearer to* the observer in (a) the spatial and (b) the temporal tasks**.

Direction of FRONT	Country			
	Germany	USA	China	Tonga
*(a) Space*^1^	(*N* = 69)	(*N* = 66)	(*N* = 32)	(*N* = 50)
Further	10.9	22.7	43.7	73.0
Nearer	89.1	77.3	56.3	27.0
*(b) Time*^2^	(*N* = 120)	(*N* = 144)	(*N* = 163)	(*N* = 120)
Further (futurewards)	10.0	50.0	3.7	55.8
Nearer (pastwards)	90.0	50.0	96.3	44.2

*^1^Data from Beller et al. (under review), frontal condition, two tasks with non-intrinsic objects arranged in one line*.

*^2^Data from Bender et al. (2010, p. 299), event in the future*.

Yet, even these findings cannot count as conclusive evidence against a close link between spatial and temporal references. The spatial data used for this comparison was collected with table-top stationary objects, whereas the temporal data originate from the interpretation of where to an event is moved. This implies a crucial difference between the two settings: while the first setting is *static*, the second is *dynamic*. At least for USA-English, however, there is some evidence that people’s preferences may shift from static to dynamic settings (Hill, 1978, 1982; for a theoretical distinction of static and dynamic settings, see also Tenbrink, 2011). To solve this issue and assess the extent to which the FoRs underlying the spatial reading of “moving forward” also affect its temporal reading, we decided to compare people’s responses in a spatial and a temporal task *both* of which are dynamic.

## Experiment

The experiment consisted of two parts. The goal of *Part 1* was to scrutinize whether preferences for spatial FoRs in any of the four languages under scrutiny (i.e., German, USA-English, Mandarin Chinese, and Tongan) change if speakers refer to dynamic instead of static spatial constellations. Comparing this new data with the one reported in Table 2 allows us to assess whether the correspondence between spatial and temporal preferences increases if the conditions under which they are elicited are more equivalent (dynamic settings).

*Part 2* aimed at examining which reading of *moving forward* speakers of USA-English prefer in spatial as contrasted to temporal contexts. English is the one language in our sample that provides the exact same vocabulary (“moving X forward”) for spatial and temporal expressions, and whose speakers exhibit substantial intra-linguistic variance in their adoption of FoRs both in spatial and temporal tasks (Beller et al., under review; Rothe-Wulf et al., under review). Assessing to what extent individual readings of “moving X forward” co-vary across space and time is thus particularly promising for our US participants: will they adopt the same FoR to construe temporal descriptions as they do for spatial descriptions? Such a co-variation, if it occurred, would then also help to explain the inter-individual variability in the responses to the Wednesday’s meeting task found in the USA.

### Methods

#### Materials

Two types of tasks were used (four questions each), one for assessing the preferred spatial reading (s-FoR) of the verb “moving forward” (Part 1), and the other for assessing the preferred t-FoR (Part 2).

##### Part 1

In order to assess participants’ *spatial reading*, two pairs of pictures were used, each depicting one situation in a game: *Mills* (also known as *Nine Men*’*s Morris*) and *Chess*. Participants were asked to mark in the picture, where to they would move a particular game piece. For the two target pictures (see Table 4) the instruction asked to “move the front piece one position forward” (Mills) or to “move the white rook two squares forward” (Chess), respectively; the instruction also depicted a white rook to facilitate token identification. The other two pictures requested sidewards or diagonal movement and served as filler items.

**Table 4:** **Percentages of individuals choosing the further/nearer piece as “the front piece” in the Mills task [(A), bold-faced], and percentages of individuals moving the chosen piece further away from or nearer toward them [(A) Mills and (B) Chess]**.

Task	Instruction	Country			
	Move X forward	Germany	USA	China	Tonga
*(A) Mills*	*X *= *the front piece*	(*n* = 134)	(*n* = 108)	(*n* = 59)	(*n* = 66)
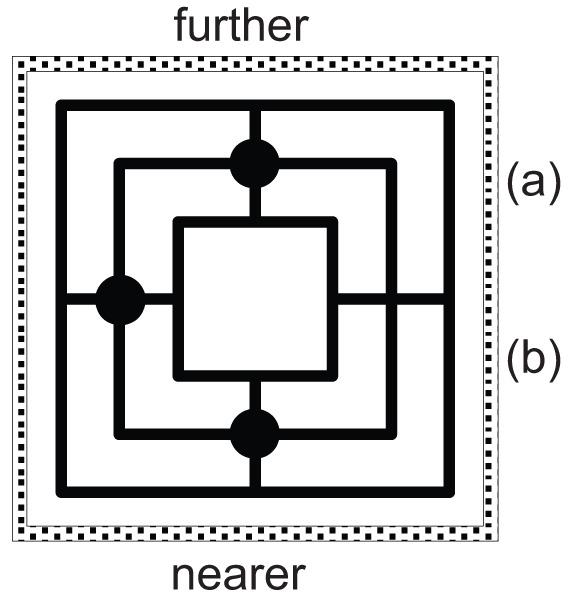	*(a) X *= *further piece*	**25.4**	**43.5**	**79.7**	**90.9**
	*Further*	24.6	34.3	76.3	66.7
	*Nearer*	0.8	9.2	3.4	24.2
	*(b) X *= *nearer piece*	**74.6**	**56.5**	**20.3**	**9.1**
	*Further*	50.7	38.9	8.5	7.6
	*Nearer*	23.9	17.6	11.8	1.5
*(B) Chess*		(*n* = 137)	(*n* = 136)	(*n* = 62)	(*n* = 74)
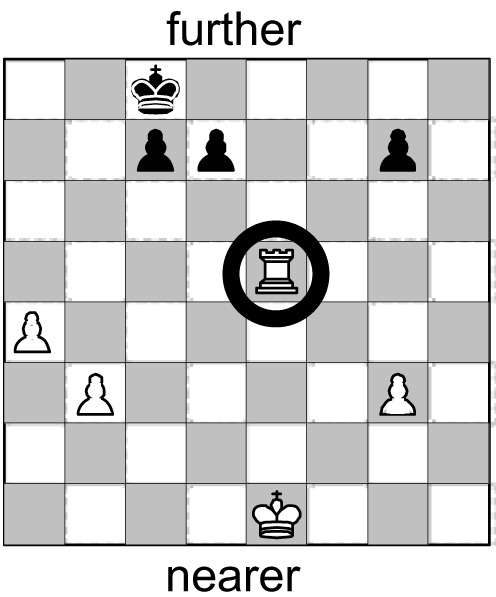	*X *= *the white rook*				
	*Further*	83.9	90.4	85.5	91.9
	*Nearer*	16.1	9.6	14.5	8.1

Mills and Chess differ in one crucial aspect, namely their inherent orientation: as Mills is played by placing round tokens alternatively on any node of the grid, anywhere on the board, both the tokens and the board lack an intrinsic front. In contrast, Chess more explicitly resembles a combat game in which – at least in the beginning – both sides are opposed to each other and in which some tokens such as the pawns have a predefined moving direction (i.e., toward the other side of the board). Furthermore, in depictions of Chess constellations, the white side is canonically the one nearer to the observer. Contrasting these two games aimed at assessing the additional effect of such an intrinsic orientation on FoR adoption. Such an effect, however, is expected to occur only if people are familiar with the rules of the game (which we inquired after completion of the tasks). If they are *not* familiar, both depictions alike should be regarded as basically non-oriented, which would then dampen any possible effects of game orientation. Please also note that the Mills task allows us to assess the preferred s-FoR in both a static and a dynamic context at the same time: picking the “front piece” (static) requires the assignment of front as much as does “moving it forward” (dynamic; see Table 1).

All materials were presented in the participants’ native languages. The phrase “moving forward” was translated into German as *nach vorne schieben*, into Chinese as *xiàng qián yí*, and into Tongan as *teke ki mu*’*a*. These phrases use the same (or cognate) prepositions as the temporal ones, but not all of them use the same verbs. In temporal contexts, the translations for “moving forward” was identical in Chinese (*xiàng qián yí*), but different in German (*vorverlegen*) and Tongan (*matolo ki mu*’*a*)^4^.

##### Part 2 (USA only)

In order to assess the *temporal reading*, two pairs of questions of the following type were used: “The meeting scheduled for Wednesday next week will be moved forward 2 days. On which day of the week will it now take place?” Each pair of questions consisted of a *future* event and a *past* event. One pair of questions used the time scale *days of the week* with a time span of 2 days for moving the event, the other pair used the time scale *time of the day* with a time span of 3 h for moving the event (type of event, time of event, and time scale were counterbalanced). Crucially, all questions had the same structure, instantiating a ternary relation between (exemplified for Wednesday’s meeting question) ground G = Wednesday, figure F = date of rescheduled meeting, and (optional) observer’s viewpoint V = speaker’s present.

An *absolute* t-FoR is assigned when both past and future events are “moved forward” toward the future, an *intrinsic* t-FoR is assigned when they are both moved toward the past, and a *relative* t-FoR is assigned when they are moved symmetrically with regard to the subjective present (i.e., *translation* when being “moved forward” means further away toward past or future, respectively, and *reflection* when being moved closer toward the present; cf. Table 1, last two columns; and see Bender et al., 2010; Rothe-Wulf et al., under review).

#### Participants

The sample consisted of 137 German students (101 female) from Freiburg University (mean age 24.9 years, *SD* = 7.0), 137 USA students (88 female) from the Pennsylvania State University (mean age 21.1 years, *SD* = 4.3), 70 Chinese students (21 female) from Tongji University (mean age 20.5 years, *SD *= 2.1), and 116 Tongan students (68 female) from Ha‘apai High School (mean age 16.4 years, *SD* = 1.1).

#### Design and procedure

The Mills and Chess tasks were each presented blockwise, and in one of two orders. The tasks were presented in a booklet, printed one each on a page. Although participants were not instructed on how to hold the booklet when responding, the booklet itself likely normalized the direction of viewing (i.e., with the spine of the booklet to the left and the top of the page further away from the participant). The tasks reported here were part of a larger survey, in which participants first worked on referencing tasks for static settings (reported in Beller et al., under review), and then on the four dynamic tasks reported here. If carry over effects from the static to the dynamic settings were to occur, they should render the latter more similar to the former ones.

The temporal tasks were presented in the USA sample only (for the reasons given above), and *before* the spatial tasks. The latter is justified by the fact that spatial representations may affect temporal reasoning, but not the other way around (Boroditsky, 2000; Casasanto and Boroditsky, 2008; Casasanto et al., 2010).

### Results and discussion

We will first analyze the spatial data across the four countries (Part 1) and then the relation between space and time in the USA (Part 2).

#### Part 1: spatial tasks

For the analysis of the spatial data, we excluded those participants, who did not indicate unambiguously which piece they had moved (in the Mills task only^5^), and those who performed a movement to the left or to the right (in Mills or Chess). For the remaining participants, we determined whether the piece chosen was moved *further away from* or *nearer toward* them (Table 4).

The Mills task combines a static question (“which piece is chosen as the *front* piece?”) with a dynamic question (“in which direction is it moved *forward?*”). With regard to the *static* question, choosing the piece nearer to the observer as “the front piece” corresponds to the reflection (or rotation) variant of the relative s-FoR, whereas choosing the piece further away from the observer corresponds to the translation variant^6^. The preferences for one or the other piece (Table 4, percentages printed in bold) differ substantially between countries (χ^2^ = 99.1; *df* = 3; *p* < 0.001; *N* = 367) and are in line with our previous results (cf. Tables 2 and 3): in Germany, the preference for the nearer piece (further: 25.4% vs. nearer: 74.6%) is consistent with the reflection variant; the preference for the further away piece in China (further: 79.7% vs. nearer: 20.3%) and in Tonga (further: 90.9% vs. nearer: 9.1%) reveals the translation variant; and the results in the USA (further: 43.5% vs. nearer: 56.5%) indicate that the reflective reading slightly dominates the translational one.

With regard to the *dynamic* question (“in which direction is the piece moved forward?”), the picture looks quite different: here, we found no differences between countries, as indicated by a log-linear analysis (Kennedy, 1992) with “direction of movement” as dependent variable (main effect “country”: *G^2^* = 6.0; *df* = 3; *p* = 0.112). Instead, the direction of movement depended on which piece was chosen as “the front piece” (main effect “piece”: *G^2^* = 15.7; *df* = 1; *p* < 0.001) and was modulated to some extent by the country as indicated by a significant interaction (“country × piece”: *G^2^* = 16.1; *df* = 3; *p* = 0.001).

**Figure 3:**
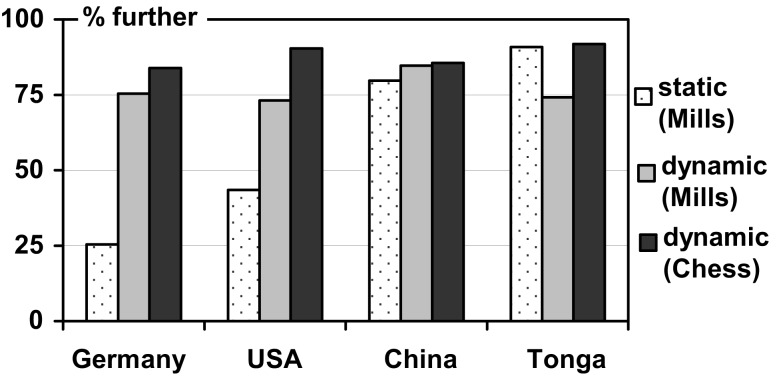
**Percentage of individuals assigning FRONT *further away* from the observer in the static and the dynamic tasks (cf. Table 4)**.

Across all four countries, we found a clear preference for a translational reading, that is, for moving the piece further away from the observer (further: 76.0% vs. nearer: 24.0%; χ^2^ = 99.4; *df* = 1; *p* < 0.001; *N* = 367). In China and Tonga, references in the dynamic setting are thus consistent with those in the static settings (both translation), whereas in the USA and particularly in Germany, they are not. Here, in line with Hill’s (1978, 1982) observations, the switch from a static to a dynamic setting was sufficient to switch the preferences from reflection to translation or from a “nearer” to a “further away” positioning (as depicted in Figure 3). Overall, the preference for the translational reading was even stronger for participants who had chosen the piece further away according to the translation variant (further: 84.6% vs. nearer: 15.4%; *n* = 188) than for participants who had chosen the piece nearer toward them (further: 67.0% vs. nearer: 33.0%; *n* = 179). This indicates at least a tendency for being consistent in the static and dynamic aspect, which varies, however, between the four countries: it is strongest in China (88.1% consistent choices), followed by Tonga (68.2%), the USA (51.9%), and Germany (48.5%). In other words, roughly half of the participants in the USA and Germany applied different spatial FoRs for static as opposed to dynamic settings.

The Chess task entails only the dynamic aspect (i.e., the direction in which the piece is moved), but no static aspect. On the other hand, it allows us to assess an additional effect of an intrinsic front assigned to the token. As noted above, the two settings differ in that Chess does, but Mills does not contain an inherent orientation; being white, the rook depicted in this task is intrinsically oriented toward the side of the black tokens. Similar to the Mills task, we found a strong preference for a reading as further (87.8%) over nearer (12.2%; χ^2^ = 233.5; *df* = 1; *p* < 0.001; *N* = 409) with no differences between countries; χ^2^ = 4.2; *df* = 3; *p* = 0.236; *N* = 409 (see Table 4). Presumably due to the intrinsic orientation of the white rook, this reading is even more pronounced than in the Mills task^7^.

**Figure 4:**
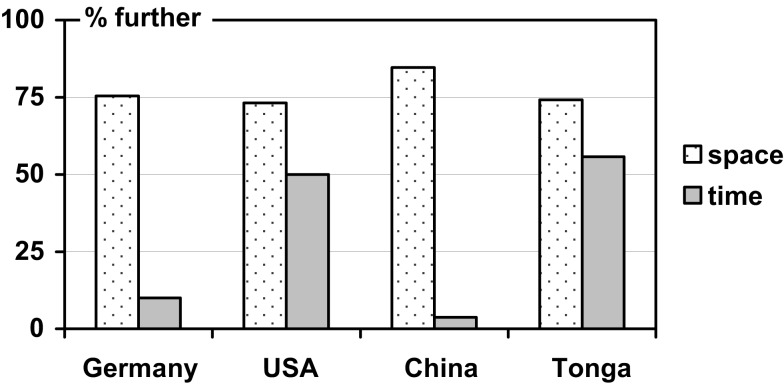
**Percentage of individuals assigning FRONT *further away* from the observer in space (Mills task, cf. Table 4) and time (cf. Table 3)**.

Across both tasks, speakers of all four languages generally preferred the *same* s-FoR in the dynamic settings: translation. This immediately reveals that the correspondence between preferences for spatial and temporal FoRs has not increased by making the conditions more similar. To the contrary: with Germany and China, we now have two cases with just opposite preferences for assigning front in spatial and temporal movements: further away from the observer in space and nearer toward the observer in time (see Figure 4 in comparison to Figure 2). In the USA, the preference in spatial tasks has changed from “nearer” to “further”; only the Tongan pattern, while exhibiting a significant difference, does not accumulate to an inversion of preferences^8^.

#### Part 2: temporal tasks

As a last resort for establishing cross-domain consistency, we tested co-variation of preferences for s-FoR and t-FoR on an *individual* level among our USA participants. Do individual speakers, who read the spatial “moving forward” of objects as *further away* from themselves (and thus as translational), also prefer the translational reading in time, and vice versa? As we have seen above, our USA participants predominantly chose the *translational* FoR in the spatial dynamic tasks. In contrast, the vast majority of FoRs adopted in the temporal tasks split almost evenly between *absolute* (38%) and *intrinsic* (36%), as defined in Table 1 (i.e., participants either gave pastwards or futurewards responses, respectively^9^).

While this latter finding is entirely in line with the general pattern as documented in the literature (e.g., McGlone and Harding, 1998), the underlying classification of response patterns into (t-) FoRs may not be unequivocal. As on page 4 above, we therefore also tested the simpler question of whether individual speakers, who read the “moving forward” of objects as *further away* from themselves, also prefer the futurewards reading in time (event moved further away). To answer this question, we analyzed the correspondence between participants’ moving direction in the Mills task (with the spatial array in the person’s visual field) and in two of the temporal tasks (those with an event in the future). If people choose front consistently across space and time, we would expect a high proportion of futurewards movements (further away from present) for the group of participants (*n* = 79) who made a (translational) movement further away from observer in the spatial task, and a high proportion of pastwards movements (nearer toward present) for the group of participants (*n* = 29) who made a (reflective) movement nearer toward observer in the spatial task. The data, however, do not support this hypothesis: the two groups did not differ in the mean frequency of pastwards (nearer) movements in the temporal task [53.8 vs. 50.0%; *t*(106) = 0.376, *p* = 0.707], and the correlation between spatial and temporal movement directions is close to zero (*r* = −0.037, *p* = 0.354, *N* = 108). In other words, even if the tasks are made as similar as possible to each other, the FoRs adopted for space and time (at least in the USA) appear to be entirely independent of each other.

## General Discussion

The prime goal of this study was to examine whether the preferences for a specific FoR in spatial contexts would carry over to the temporal domain. Given the large body of research attesting to the link between space and time, we expected this to be the case (cf. Bender et al., 2010).

Our current findings, however, are rather discouraging in this regard. Not only did we find no correspondence between temporal and spatial references in the four languages under scrutiny, we did not even find a hint of correlation in the one case that was most promising, USA-English. In this language, the very same phrase (“moving X forward”) can be used to construct similar spatial and temporal tasks; and in both domains, this phrase gives rise to considerable inter-individual variability due to its inherent ambiguity (Beller et al., under review; Rothe-Wulf et al., under review). In addition, the USA participants even worked on the temporal and the spatial task consecutively. If anything, then this should have made set effects more likely, thus increasing – at least slightly – the homogeneity in FoR adoption across domains. And yet, their spatial and temporal readings of “moving X forward” did not co-vary at all.

Again, we cannot entirely exclude that our taxonomy of temporal FoRs is inappropriate, despite its thorough conceptual grounding. In order to address respective doubts regarding how we categorized response patterns into t-FoRs, we therefore re-coded the responses according to the simpler distinction in moving directions (nearer/further). But still, the lack of consistency across domains persists.

This finding not only contradicts our own expectations, but also appears to be in contrast to the findings reported in the introduction according to which representations of space and time do interact, sometimes in rather intricate and complex manners (e.g., when watching the moving of squares on a screen affects responses to temporal tasks). So, why do we find no carry-over from space to time in this rather simple case?^10^

Several reasons are conceivable. One could be that the spatial and temporal settings used in our tasks still differ in crucial aspects. For instance, moving a game token one or two positions forward surely constitutes a small-scale setting, whereas moving an event like a meeting or flight departure forward by hours or even days might be regarded as a large-scale setting, and people are well known to be sensitive to such distinctions (Bennardo, 2000; Levinson, 2003).

Another questionable assumption regarding comparability is whether temporal ground objects can be conceived of as having an intrinsic front or not (and opinions in this regard differ largely among scholars; e.g., Bender et al., 2010; Yu, 2012; vs. Zinken, 2010; Tenbrink, 2011). This is related to the concern that the orientation inherent specifically in our Chess task may have overshadowed the patterns otherwise to be expected in the spatial tasks (i.e., prevailingly a relative FoR). We do think that this is partly the case (and this was why we contrasted a non-directional game like Mills with a directional one like Chess in the first place). However, the comparison of the Mills and the Chess task, and specifically the lack of substantial differences between the two tasks, encourages us to interpret the data of these two tasks indeed as indicative of a relative FoR. But clearly, this hypothesis calls for further investigation in future research.

Previous studies that explored the culture-specificity of cross-domain mapping targeted (non-Western) speech communities with a documented preference for the absolute FoR in spatial contexts. Setting absolute FoRs in contrast to the intrinsic and/or the relative FoRs arguably resembles a more coarse-grained investigation of this mapping than our investigation that embraced all possible FoRs. It could thus be, as was argued by one of the reviewers for this paper, that spatial and temporal conceptions may simply not map thoroughly enough to produce co-variation at this level of inspection. Given the range of both static and dynamic settings mustered for our comparison and the variety of response coding (both as FoRs and as simple further/nearer direction), it remains puzzling, though, that absolutely no co-variation emerged for the USA participants, whose temporal references do co-vary with different – and occasionally superficial – manipulations (e.g., Boroditsky and Ramscar, 2002; Kranjec, 2006; Núñez et al., 2006; Weger and Pratt, 2008).

Another reason for the observed lack of cross-domain consistency could be that (cultural) preferences for one reading over the other may arise differently for different domains. Just as speakers of closely related languages come up with different FoR preferences for disambiguating the same underspecified phrase (Rothe-Wulf et al., under review), so may speakers of one and the same language come up with different FoR preferences for the same phrase in different contexts and/or domains. Given that assignment of front for underspecified phrases is always an arbitrary act – depending on the perspective one takes – other cultural factors may simply override a tendency toward cross-domain consistency (if such a tendency ever existed in the first place). The observation that preferences do switch from reflection (in static) to translation (in dynamic) tasks in Germany and the USA lends some empirical support to this assumption.

This would also help to explain, at least to some extent, the discrepancy between other studies and our own regarding cross-domain consistency in FoR preferences. Let’s assume that preferences for FoRs do differ for space and time and do not normally carry over across domains. If a task then demands to solve temporal references, people are likely to adopt that FoR they typically prefer in such cases. If, on the other hand, the task requires a response that contains not only a temporal, but also a spatial dimension, then spatial FoRs need to be considered as well. For instance, the co-speech gestures documented by Núñez and colleagues (Núñez and Sweetser, 2006; Cooperrider and Núñez, 2009; Núñez et al., 2012) or by Le Guen and Pool Balam (2012) are necessarily spatial in nature, regardless of the domain they refer to – and this apparently poses a problem to the Yucatec Maya, who do *not* intend to indicate spatial meaning when talking about time (cf. Le Guen and Pool Balam, 2012). Likewise, abstract pointing and card arrangement tasks (as used, e.g., by Boroditsky and Gaby, 2010; Brown, 2012; Gaby, 2012) also contain a spatial dimension. In all these cases, the cross-domain consistency (if it occurred) could be attributed to this shared spatial dimension. In other words: the FoR preferences exhibited by the responses in the (temporal) tasks would then be consistent with the FoR preferences in spatial contexts simply because the spatial aspects of the response follows from the conventions of the spatial domain only. In contrast, the FoR adopted for disambiguating a temporal expression (as in our study) follows from the conventions of the temporal domain, which could be independent of the spatial ones. A similar conclusion has been drawn in a recent review on the comparison of Mandarin and English which argues that the relationship between spatial and temporal languages and reasoning is a rather complex one, and one that varies with a range of factors (Chen and O’Seaghdha, in press).

A stronger version of the above argument (that FoR preferences may be domain- and perhaps even task-specific) would be to claim that the speakers of the languages under scrutiny here do not adopt any FoR, but simply follow linguistic conventions engrained in, or contributing to, the semantics of the words – a claim often raised in discussions on these issues. However, if this was true, this argument should hold for spatial as much as for temporal contexts. The whole concept of FoRs and each concern with it would then be entirely meaningless. The very fact that the reading of phrases like “moving forward” differs across speakers, tasks, and settings – in other words: that speakers seem to change their reading upon the slightest modification of boundary conditions – justifies the assumption that they in fact do switch perspectives which, in turn, indicates that they do adopt a FoR, in time as much as in space.

Given the evidence against the use of corresponding FoRs across domains, should we continue to put effort into our attempts to generate a systematic mapping of one onto the other? We are convinced that the current findings render this endeavor indeed even more important. The conceptual link between these two domains appears to vary across levels of representation and processing. Cross-domain comparisons could help to assess at which level, to what extent, and under which conditions preferences for FoRs in space are also reflected in time. Such cross-domain comparisons, however, presuppose a consistent and comprehensive mapping of FoR taxonomies, which therefore remains one of the crucial preconditions for moving forward in this field of research.

## Conflict of Interest Statement

The authors declare that the research was conducted in the absence of any commercial or financial relationships that could be construed as a potential conflict of interest.

## Footnotes

^1^Some scholars classify the spatial orientation encoded in writing/reading direction as an example of an absolute FoR (e.g., Kranjec, 2006), but as this direction is not used to organize spatial references more generally, it is not considered here.

^2^Please note that this type of rotation (of the observer around his or her own axis towards the object array) is different from the rotation variant of the relative FoR, in which the coordinate system is transferred from V into G, by way of rotating it in G.

^3^Except for Germany, the proportion of “further” responses in the temporal tasks is significantly different from the proportion of “further” in the spatial tasks (Germany: *p* = 447; all other countries: *p* < 001; according to the binomial distribution).

^4^Of course, when people are aware of the ambiguity in these terms, they may choose unambiguous expressions such as “moving X to an earlier or later date”.

^5^In the Mills task, some participants either had given no answer or had marked the center of the board without indicating the piece that had been moved.

^6^One might also argue that identifying the figure F in this task could be resolved with an *intrinsic FoR* that originates in Ego (as ground G), with the “front piece” simply being “the piece in front of Ego”. However, as there is more than one piece involved – all of which are “in front of Ego” and thus qualify as front pieces in this sense – disambiguating the one that is *more* in front requires consideration also of the relation between these different pieces, which renders the group of non-figure pieces the ground. In consequence, the relation under scrutiny is a ternary relation and therefore requires the relative FoR.

^7^Of course, the intrinsic orientation in Chess can only come to bear on FoR choice if it is known, but this was the case in almost all samples. In contrast, the lack of acquaintance with the Mills task, in all but the German sample, should have no effect, as an intrinsic reading is not possible in either case. And indeed, the German results for the dynamic subtask do not differ significantly from the results of the other groups (χ^2^ = 3.1 *df* = 3; *p* = 377; *N* = 367).

^8^In all four countries, the difference between the frequency of the “further” response in the temporal and the spatial tasks are statistically significant (Germany, USA, and China: *p* < 001; Tonga: *p* = 017; Fisher’s exact test, 2-tailed).

^9^The relative FoRs prevailing in the spatial tasks are adopted (in a consistent manner) only exceptionally in temporal tasks (reflective FoR: 5%; translational FoR: 1%) – which defies any possibility for cross-domain correspondence.

^10^What renders matters even more disturbing is the fact, that even when priming the spatial FoRs, we had difficulties to obtain any effect on how US participants responded to the “moving Wednesday’s meeting” task (Rothe, Beller, and Bender, submission).
